# ATP-Induced IL-1β Specific Secretion: True Under Stringent Conditions

**DOI:** 10.3389/fimmu.2015.00054

**Published:** 2015-02-12

**Authors:** Monique Stoffels, Ruben Zaal, Nina Kok, Jos W. M. van der Meer, Charles A. Dinarello, Anna Simon

**Affiliations:** ^1^Department of Medicine, Radboud University Medical Center, Nijmegen Institute for Infection, Inflammation and Immunity (N4i), Nijmegen, Netherlands

**Keywords:** interleukin-1, ATP, active secretion, cell death, stringent experimental conditions

## Abstract

Interleukin-1β is a potent proinflammatory cytokine, of which processing and secretion are tightly regulated. After exposure to various stimuli, mononuclear phagocytes synthesize the inactive precursor (pro-IL-1β), which is then cleaved intracellularly by caspase-1 and secreted. A widely used method for *in vitro* secretion of IL-1β employs LPS-primed human peripheral blood monocytes. Subsequently, adenosine triphosphate (ATP) is added to the cells in order to trigger the P2X7 receptor resulting in processing and secretion of mature IL-1β. However, it is often reported that secretion is due to cytotoxic effects of ATP with P2X7 receptor-activation-related cell death. We have challenged this concept and demonstrate IL-1β specific secretion, since there is no increase in cell death and IL-1α and IL-18 are not released in the same cultures. More importantly we show that these conclusions can only be drawn under stringent experimental conditions.

## Introduction

IL-1β is a potent cytokine able to induce inflammation. It has a role in infection, diabetes, heart failure, and neuro-inflammatory diseases (1, 2) as well as several autoinflammatory diseases.

It is a product of monocytes/macrophages, dendritic cells, natural killer-cells, and B-lymphocytes (1). When stimulated with pathogen associated molecular patterns, such as LPS, or damage associated molecular patterns, these cells produce inactive pro-IL-1β with a molecular weight of 31 kDa (1, 3), which accumulates in the cytosol. As mature IL-1β lacks a signal peptide, its secretion was enigmatic until crucial experiments done by Rubartelli shedded light (4); when a second signal, such as extracellular adenosine triphosphate (ATP), activates the cation-selective P2X7 receptor on the cell membrane, a potassium efflux occurs, via a pore permeable to hydrophilic solutes up to 900 Da (5–7). This fall of intracellular potassium triggers assembly of the inflammasome protein complex, which in turn leads to production of active caspase-1. Activated caspase-1 cleaves the precursor of IL-1β into an active 17 kD form, which is secreted (1, 8). Several reports showed that specific polymorphisms in P2X7 impair its function (9–12) and as a result, IL-1β secretion is reduced, suggesting a vital role for P2X7 in IL-1β secretion (13).

In a pioneering study, Ferrari et al. (8) described a model now widely used to study IL-1β release, which consists of brief LPS-stimulation for induction of pro-IL-1β (called “priming”), followed by ATP stimulation of PBMCs to enhance secretion (8, 14–16). Even with purified LPS-stimulation alone, ATP from endogenous source is thought to be involved (17). Since IL-1β lacks a signal sequence for compartmentation within the Golgi and release through classical secretory vesicles, non-classical mechanisms are required for its secretion, which were recently reviewed by Dubyak (18). These include the secretory lysosome (containing accumulated IL-1β) pathway, the release of membrane-delimited microvesicles (surface membrane evaginations that entrapped cytosolic IL-1β) [Ref. (19, 20) and reviewed in detail in Ref. (21)], the release of membrane-delimited exosomes that occurs after the formation of multivesicular bodies by recycling endosomes that have entrapped IL-1β (22), and exocytosis of autophagosomes or autophagolysosomes.

Alternatively, it has been suggested that activation of the P2X7 receptor leads to cell death and that IL-1β is passively released with loss of membrane integrity rather than actively secreted (15, 23, 24). In the present report we investigated this issue.

## Materials and Methods

### *In vitro* cytokine production

PBMCs obtained from venous blood or buffy coats from healthy volunteers were isolated using Ficoll gradient separation. This research was approved by the local ethics committee and volunteers gave written informed consent.

#### Media and ATP solutions

RPMI 1640 medium (Invitrogen, Paisley, UK) supplemented with 1 mM sodium pyruvate (Sigma-Aldrich), 2 mM l-Glutamine (Merck) and 50 μg/ml gentamicin was used. No serum was added. For preparing ATP solutions, the following directions were carefully followed. First, the amount of ATP (Sigma, A6419) needed for the experiment was weighed in an Eppendorf tube. Calculations and solutions (excluding ATP) were all prepared beforehand. At the desired time of preparation (fresh, or *x* minutes before adding to the plates), the ATP in the Eppendorf tube was dissolved in medium to generate a 100 mM stock and was immediately further diluted into the pre-pipetted tubes yielding the final ATP concentrations. ATP solutions were immediately added to the cells. This had to be done as fast as possible to make sure that the time between preparation of the ATP solution and adding this to the cells was 2 min (or as otherwise indicated in the figures). Usage of automated multi-channel pipets is highly recommended. All solutions were prepared from medium pre-warmed at 37°C and kept in the laminar flow cabinet, with an inside temperature of 21°C, until adding them to the cells.

#### Experimental procedure

Hundred microliter of cells at a concentration of 5.0 × 10^6^/ml was pipetted into 96-wells U-bottom plates (Greiner 650180) and cells were exposed to LPS for 24 or 3 h [Sigma, *Escherichia coli* 055:B5, purified as described (25)] in a total volume of 200 μl. For the 3 h incubation in combination with ATP we added 100 μl of medium or medium containing 2 μg/ml LPS to 100 μl of cells, resulting in a final LPS concentration of 1 μg/ml for a total of 500,000 cells in each well. Samples were prepared in duplicates. After 3 h of incubation at 37°C, the medium was removed from the cells by careful pipetting. Then 200 μl of medium, or ATP in medium was added and incubated for exactly 15 min at 37°C, after which cells were spun down at 350 g for 8 min at room temperature. One hundred fifty microliter supernatant per well was collected and pooled with its duplicate, such that in total 300 μl supernatant per condition was collected. The remaining 50 μl supernatant was discarded because this may have contained cell material. Hundred microliter of medium (same as used for the incubation) was added to the remaining cells, and plates were subjected to three cycles of freeze-thawing. Plates were spinned as described above and 80 μl supernatant was collected and pooled with its duplicate for cell-associated cytokine analysis. To obtain experimental replicates, we repeated the experiments with different donors and at various days. Cytokine concentrations were measured using ELISA (R&D systems; IL-1α, total IL-1β, and specific intact pro-IL-1β) or Luminex (Bio-Rad; IL-18). Detection limits for IL-1β, IL-1α, and pro-IL-1β ELISAs were 39, 39, and 70 pg/mL, respectively.

### Cell viability assays

Cell viability was assessed by two independent LDH assays; in supernatants measured by ARCHITECT C16000 system (Abbott Laboratories, USA), and measured in phenol-red free supernatants by CytoTox96 Non-Radioactive cytotoxicity assay according to manufacturer’s instructions (Promega, Madison, USA). In the latter, stimulated samples were compared to the non-stimulated samples (viable controls); lysed cells from the same donors served as positive controls. In addition, we employed trypan blue staining (Sigma, St. Louis, USA) and an AnnexinV-PI staining according to the manufacturer’s recommendations (Biovision, Milpitas, USA). In brief, after the indicated stimulations, cells were resuspended in AnnexinV-FITC Staining solution and incubated in the dark for 15 min on ice. PI was added and cells were incubated in the dark for another 5 min on ice. The cells were measured on a FC500 flow cytometer (Beckman Coulter) and the data were analyzed using CXP analysis software v2.2 (Beckman Coulter).

### qRT-PCR

Total RNA was extracted from PBMCs using TRIzol reagent (Invitrogen), subjected to DNAse treatment (Ambion^®^ DNA-free™ Kit, Invitrogen) and reverse-transcribed into cDNA (iScript cDNA Synthesis Kit, Bio-Rad). qRT-PCR was performed using an Applied Biosystems 7300 real-time PCR using the following primers: IL-1β: 5′-CAGCTACGAATCTCCGACCAC-3′ (forward) and 5′-GGCAGGGAACCAGCATCTTC-3′ (reverse); β2M: 5′-ATGAGTATGCCTGCCGTGTG-3′ (forward); and 5′-CCAAATGCGGCATCTTCAAAC-3′ (reverse).

### Statistical analysis

Results were analyzed using the Wilcoxon matched-pairs signed rank test for non-normally distributed paired data, in GraphPad Prism, version 5.00 (GraphPad Software, San Diego, CA, USA). ***p ≤ 0.001; **p ≤ 0.01; *p ≤ 0.05.

## Results

**Figure 1:**
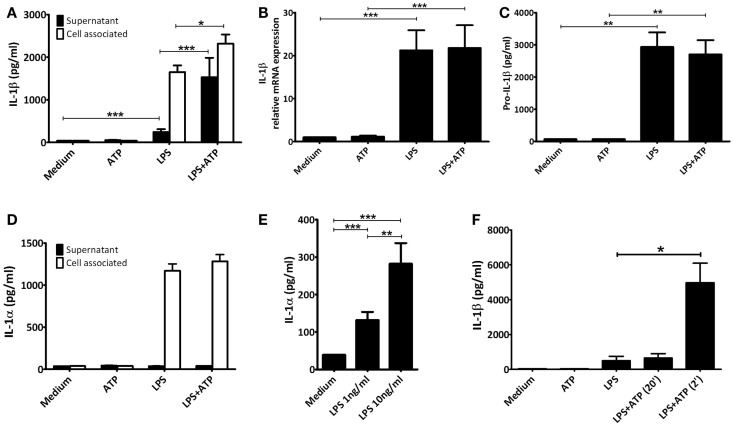
**IL-1 concentrations**. PBMCs were incubated with either medium or 1 μg/ml LPS for 3 h, and additionally with medium or 1 mM ATP for 15 min. All cytokines were measured in the same preparations (*n* = 12). **(A)** Significant increased IL-1β levels after stimulating LPS-primed PBMCs with 1 mM ATP compared to LPS-priming only. **(B)** LPS-induced IL-1β mRNA expression. **(C)** Intracellular pro-IL-1β levels. **(D)** LPS-induced intracellular IL-1α concentrations. No IL-1α could be detected in supernatants. **(E)** PBMCs incubated for 24 h with 1 or 10 ng/mL LPS (*n* = 16) secrete significantly more IL-1α than unstimulated PBMCs, in a dose-dependent manner. **(F)** Comparison of IL-1β secretion after adding ATP 2 or 20 min after preparation (*n* = 6). IL-1β levels were determined in a separate experiment, using the same setup. All data are represented as mean + SEM. IL-1β levels detected in **(A,F)** represent both mature and pro-IL-1β, whereas in **(C)** specifically intact pro-IL-1β levels were detected.

In human PBMCs priming with LPS alone for 3 h led to IL-1β secretion (mean 0.25 ng/mL ± 0.07 SEM, *n* = 12), which was significantly elevated when followed by ATP exposure (mean 1.53 ng/mL ± 0.46 SEM, *p* < 0.001, *n* = 12) (Figure 1A). Secretion of another IL-1 family member cleaved by caspase-1, IL-18, was not increased (IL-18 concentration in supernatant in all samples below level of detection). Upon LPS-priming, IL-1β mRNA expression increased 21-fold. However, ATP stimulation did not further influence the IL-1β mRNA expression (Figure 1B). Priming also induced the expression of intracellular pro-IL-1β protein (Figure 1C). The additional pulse with ATP had no effect on these concentrations. There was an increase in intracellular total IL-1β concentration after ATP (Figure 1A).

We assessed cell viability by trypan blue staining and LDH release. In our experiments, neither of the stimuli increased cell death, compared to unstimulated samples (data not shown). In addition, though LPS-priming induced production of IL-1α this was not secreted into the supernatant, not even in the presence of ATP (Figure 1D). After 24 h of stimulation with either 1 or 10 ng/mL LPS however, cells were secreting IL-1α, in a dose-dependent manner (means 132 pg/mL ± 22 SEM, and 282 pg/mL ± 55 SEM, respectively, *n* = 16) (Figure 1E). This was not due to cell death, as LDH remained below the detection limit (data not shown).

The ATP stimulus is crucial for actual secretion of IL-1β *in vitro*. Therefore, we tested the influence of the quality of the ATP solutions. Dissolving ATP in RPMI 2 min before addition to the cells resulted in a significant increased IL-1β secretion. If ATP was dissolved 20 min before addition, the effect was completely abrogated (Figure 1F).

**Figure 2:**
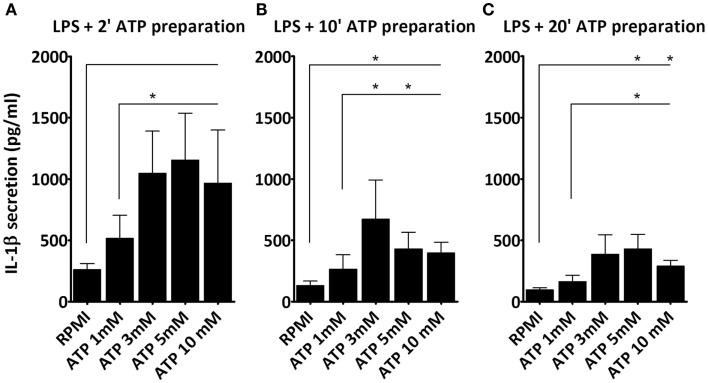
**Effect of ATP preparations on IL-1β secretion**. PBMCs were incubated with either medium or 1 μg/ml LPS for 3 h, and additionally with medium or different concentrations of ATP for 15 min. We observed a dose dependent increase in IL-1β secretion regardless of the time in between the ATP preparation and the stimulation, with a maximum response at a dose of 3 mM ATP. IL-1β secretion was higher when fresh ATP preparations were used **(A)**, as compared to older preparations, e.g., 10 min **(B)**, and 20 min old **(C)**. Results from two independent experiments with a total of six donors were pooled and represented as mean + SEM.

Next, we studied whether the ATP concentration could overcome the diminished stimulatory capacity of the “older” ATP preparation. As shown (Figure 2), cells stimulated with LPS and subsequently with ATP that was prepared 2 min (Figure 2A), 10 min (Figure 2B), or 20 min (Figure 2C) before addition, all secrete IL-1β. However, IL-1β secretion decreased with increasing time in between the preparation and the stimulation with ATP. Also, at each time point 3 mM ATP induces a greater IL-1β secretion than 1 mM ATP. However, the 5 and 10 mM concentrations do not increase the response further. Thus, the maximum response in this cell model is observed at 3 mM ATP. Nevertheless, even this response is not able to overcome the preparation effect; with a 20 min 3 mM ATP preparation IL-1β secretion is still lower than with a 2 min 1 mM ATP preparation.

**Figure 3:**
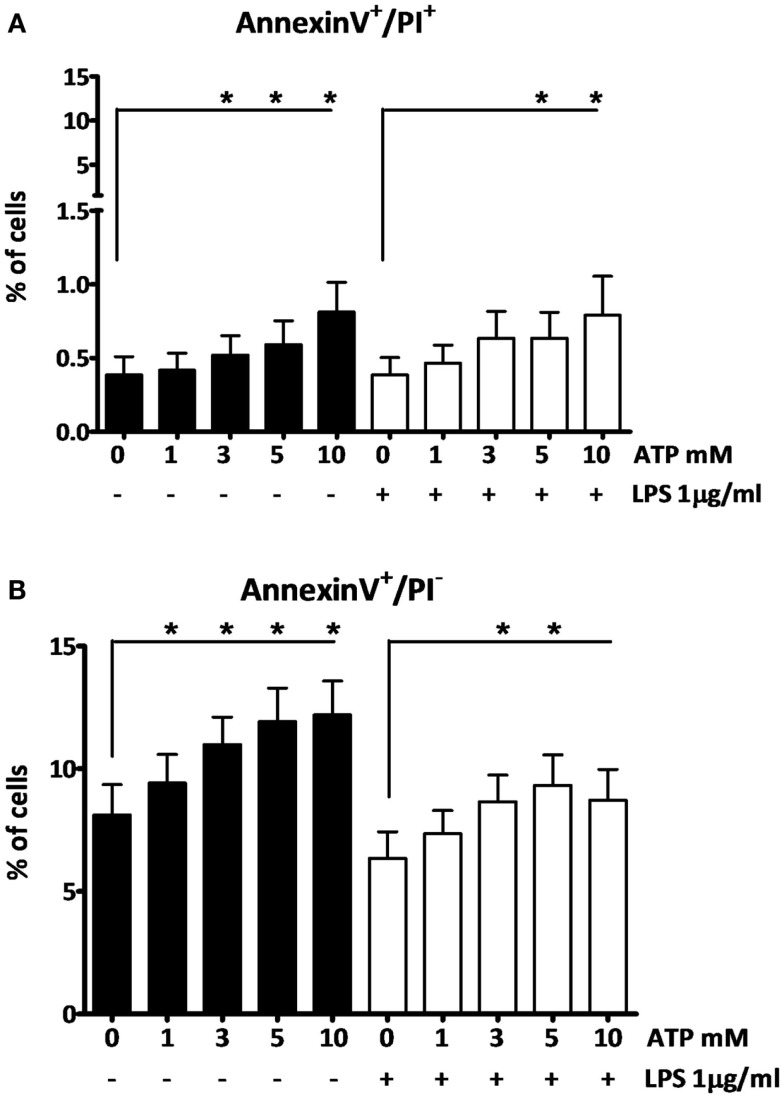
**The effect of fresh ATP preparations on cell membrane integrity**. **(A)** Cells that lost membrane integrity stain positive for AnnexinV and PI, and can be late apoptotic or necrotic. **(B)** Cells that stain only positive for AnnexinV are early apoptotic but have not lost membrane integrity. Higher concentrations of ATP cause more cell death **(A)** than a low concentration. ATP induces early apoptosis in all concentrations **(B)**. Results from two independent experiments with a total of six donors were pooled and represented as mean + SEM.

To investigate the effects of the different ATP concentrations on cell viability we further used AnnexinV-PI staining. At the early stages of apoptosis, cells start exposing phosphatidylserine (PS) to the external cellular environment, making them positive for AnnexinV-FITC staining. Loss of membrane integrity in late apoptotic or necrotic cells renders them permeable to PI. After 3 h LPS followed by 15 min ATP stimulation, only higher ATP concentrations (≥3 mM) induce cells to become late apoptotic or necrotic (AnnexinV^+^/PI^+^), although only at a low percentage (Figure 3A). Early apoptotic cells (AnnexinV^+^/PI^−^) were observed at all ATP concentrations (Figure 3B). In the absence of LPS cells were more sensitive to the ATP stimulus, than in the presence of LPS, as indicated by the black bars and white bars, respectively (Figure 3).

## Discussion

We confirmed earlier findings (8, 15, 16) that PBMCs from human volunteers secrete significantly more IL-1β when primed with LPS for 3 h followed by a pulse with ATP for 15 min compared to LPS-stimulation alone (Figure 1A). This difference is not due to increased mRNA expression (Figure 1B) or an increase in the intracellular level of pro-IL-1β (Figure 1C). This confirms that ATP-induced P2X7 activation leads to caspase-1 activation, and the processing of pro-IL-1β and secretion of the mature molecule. It is interesting to note that the total levels of IL-1β are higher, while intracellular pro-IL-1β levels remain similar when comparing the LPS and LPS + ATP stimulation. This may be explained by a simultaneous increase in translation efficiency and cleavage of pro-IL-1β under influence of ATP. In addition, Levandowski et al. reported that a SNP in NLRP1 results in increased IL-1β secretion, while pro-IL-1β levels remained similar (26). Alternatively, on the total levels of IL-1β, the percentage of secreted IL-1β is very low compared to the percentage of pro-IL-1β present in the cells, and it is therefore possible that a significant decrease in pro-IL-1β would not be observed.

There are a number of controversies or uncertainties regarding the effects of ATP in this setting. First, whether the effect is due to cell death or not. Ferrari et al. observed no increase in cell death after exposure of LPS-primed macrophages to ATP (8), while Brough et al. did report ATP increased cell death (15) as evidenced by increased IL-1α and LDH (15, 27). In our experiments, 15 min incubation of 1 mM of ATP to LPS-primed PBMCs did not increase cell death as detected by LDH and AnnexinV-PI staining. Despite high intracellular concentrations of IL-1α after 3 h of LPS-stimulation (either with or without ATP pulse), we detected no increase in the supernatants (Figure 1D). This confirms that the cell membrane is intact, and it follows therefore that extracellular IL-1β concentrations are due to active secretion rather than passive release. Differences in experimental conditions in the studies mentioned (5 vs. 1 mM ATP; 30 vs. 15 min ATP incubation) (8, 15) could very well explain these discrepancies.

Secondly, is this ATP effect specific for IL-1β or does ATP facilitate the release of other cytokines from the same cell? For example, Fettelschoss et al. (28) reported that ATP is needed for secretion of IL-1α after 24 h of stimulation with LPS. We found no effect of ATP on the LPS-induced increase in intracellular IL-1α concentration (Figure 1D); and after 24 h of incubation LPS alone was sufficient to induce secretion of IL-1α (Figure 1E). This was not due to increased cell death as determined by LDH measurement (not shown). These data also do not support the hypothesis that secretion of IL-1α is coupled to secretion of IL-1β (Figures 1A,D,E) (28). We found no increased secretion of IL-18, another cytokine of the IL-1 family that is processed by caspase-1 (data not shown). Although both IL-1β and IL-18 are substrates for caspase-1, they are regulated differently (29), and since IL-1β in this assay is detected before the peak in its secretion, this may also explain why IL-18 secretion remained below our detection limit. Accordingly, the effect of ATP stimulation is specific for IL-1β.

A final controversy is that the result of the LPS/ATP model is not always consistent. For example, Ferrari et al. found that LPS-primed monocytes did not exhibit an increase in IL-1β secretion after stimulation with ATP, although macrophages did (8). Our data do show an increase in IL-1β secretion in PBMCs (largely originating from monocytes). Other studies routinely use much higher ATP concentrations (15). A crucial factor is likely to be the preparation of the ATP. As we demonstrate here, for optimal results ATP needs to be dissolved within 2 min before addition; when we prepared ATP 20 min before adding it to the cell preparations, there was no significant effect on secretion (Figure 1F). The difference of merely 18 min in preparation time completely abrogates the effect. This could be due to phosphohydrolysis of ATP, which may explain why some laboratories need to add a higher (almost toxic) total concentration of ATP to see any increased secretion of IL-1β. This higher total ATP concentration may induce cell death. In our model we have consistently used 1 mM ATP to stimulate the cells, although 3 mM is considered the optimal concentration. Our data show that cells do secrete IL-1β in response to this sub-optimal dose, which is closer to physiologically relevant levels. More importantly, at this dose, we could not observe cell death, whereas higher ATP concentrations do cause cell death (Figure 3). This is supported by results in a study of Kuhny et al. (30), in which the use of low or high dose ATP resulted in differences in ATP-induced morphological changes in mast cells as measured by flow cytometry. Here, 0.3 mM ATP led to minor increase of the forward scatter, while stimulation with 3 mM ATP induced the formation of a 2nd PI positive population with increased side scatter and smaller cell bodies. Moreover, in our experiments, even stimulation with high concentrations of ATP could not overcome the effect of the 20 min between preparation and stimulation (Figure 2). Thus, immediate preparation of ATP right before addition is of vital importance.

In summary, we confirmed the validity of the LPS/ATP model. IL-1β secretion is selective and not due to passive release by a cytotoxic effect of ATP. Moreover, our results emphasize the importance of stringent experimental conditions, i.e., using freshly prepared ATP in this model.

## Author Contributions

MS, RZ, JvdM, CD, and AS designed experiments; MS, RZ, and NK analyzed data; MS and RZ wrote the manuscript; AS obtained grant funding; and AS, JvdM, and CD edited the manuscript.

## Conflict of Interest Statement

The authors declare that the research was conducted in the absence of any commercial or financial relationships that could be construed as a potential conflict of interest.
